# Lung autotransplantation for bronchial necrosis after radiotherapy: a case report

**DOI:** 10.1186/s40792-021-01164-0

**Published:** 2021-04-01

**Authors:** Yuya Nobori, Masaaki Sato, Mizuki Morota, Yoshikazu Shinohara, Daisuke Yoshida, Takahiro Karasaki, Kentaro Kitano, Jun Nakajima

**Affiliations:** Department of Thoracic Surgery, Graduate School of Medicine, The University of Tokyo, 7-3-1 Hongo, Bunkyo-ku, Tokyo, 113-8655 Japan

**Keywords:** Lung autotransplantation, Bronchial necrosis, Radiation therapy, Mediastinal tumor

## Abstract

**Background:**

Bronchial necrosis is a rare but fatal complication after radiation therapy. Because of the anatomical complexity and rarity of this condition, determining the most appropriate management for individual patients is extremely challenging. Lung autotransplantation is a surgical technique that has been applied to hilar neoplastic lesions to preserve pulmonary function and avoid pneumonectomy. We herein report a case of bronchial necrosis secondary to radiotherapy that was treated with lung autotransplantation.

**Case presentation:**

A 46-year-old man developed broad necrosis and infection of the right bronchus secondary to previous stereotactic body-radiation therapy. This treatment was supplied close to a right hilar metastatic pulmonary tumor derived from a mediastinal malignant germ cell tumor that had been surgically resected with the left phrenic nerve. The bronchial necrosis accompanied by infection with *Aspergillus fumigatus* was progressive despite antibiotics and repetitive bronchoscopic debridement. Because of the patient’s critical condition and limited pulmonary function, right lung autotransplantation with preservation of the right basal segment was selected. An omental flap was placed around the bronchial anastomosis to prevent later complications. The postoperative course involved multiple complications including contralateral pneumonia and delayed wound healing at the bronchial anastomosis with resultant stenosis, the latter of which was overcome by placement of a silicone stent. The patient was discharged 5 months postoperatively. Three months after discharge, however, the patient developed hemoptysis and died of bronchopulmonary arterial fistula formation.

**Conclusions:**

We experienced an extremely challenging case of bronchial necrosis secondary to radiotherapy. The condition was managed with lung autotransplantation and omental wrapping; however, the treatment success was temporary and the patient eventually died of bronchopulmonary arterial fistula formation. This technique seems to be a feasible option for locally advanced refractory bronchial necrosis, although later complications can still be fatal.

## Background

Bronchial necrosis is a rare but fatal complication after radiation therapy [1–4]. Because of the anatomical complexity and rarity of this condition, determining the most appropriate management for individual patients is extremely challenging. Although surgical management should be absolutely considered in the presence of pneumomediastinum, there are no other definite standards of intervention [1]. Therefore, multidisciplinary team management is essential to optimize the treatment.

Lung autotransplantation is a surgical technique that can reportedly preserve lung function and avoid pneumonectomy in patients with hilar neoplastic lesions [5–7]. This surgery involves special techniques including lobectomy with or without segmentectomy on a back table, perfusion of the lung to be autotransplanted with organ-preserving solution to protect it from ischemic damage, and re-implantation by anastomosis of the bronchus, pulmonary artery, and pulmonary vein. Some reports have described autotransplantation adapted for hilar tumors and pneumonectomy-like syndrome [8–11]. To our knowledge, however, application of this technique to bronchial necrosis has not been reported. We herein report a case of lung autotransplantation in a patient with severe hilar bronchial necrosis secondary to radiotherapy.

## Case presentation

**Fig. 1:**
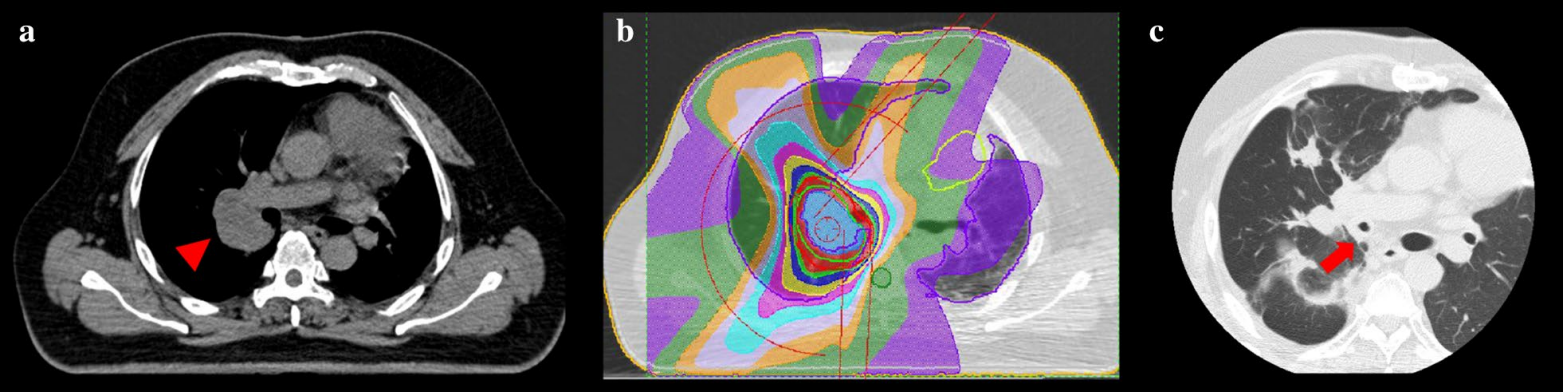
Computed tomography images before and after radiation therapy. **a** A metastatic tumor in the right hilum (red arrowhead). **b** Treatment plans for stereotactic body-radiation therapy. **c** Axial view after radiation therapy showing stenosis of the right main bronchus (red arrow)

**Fig. 2:**
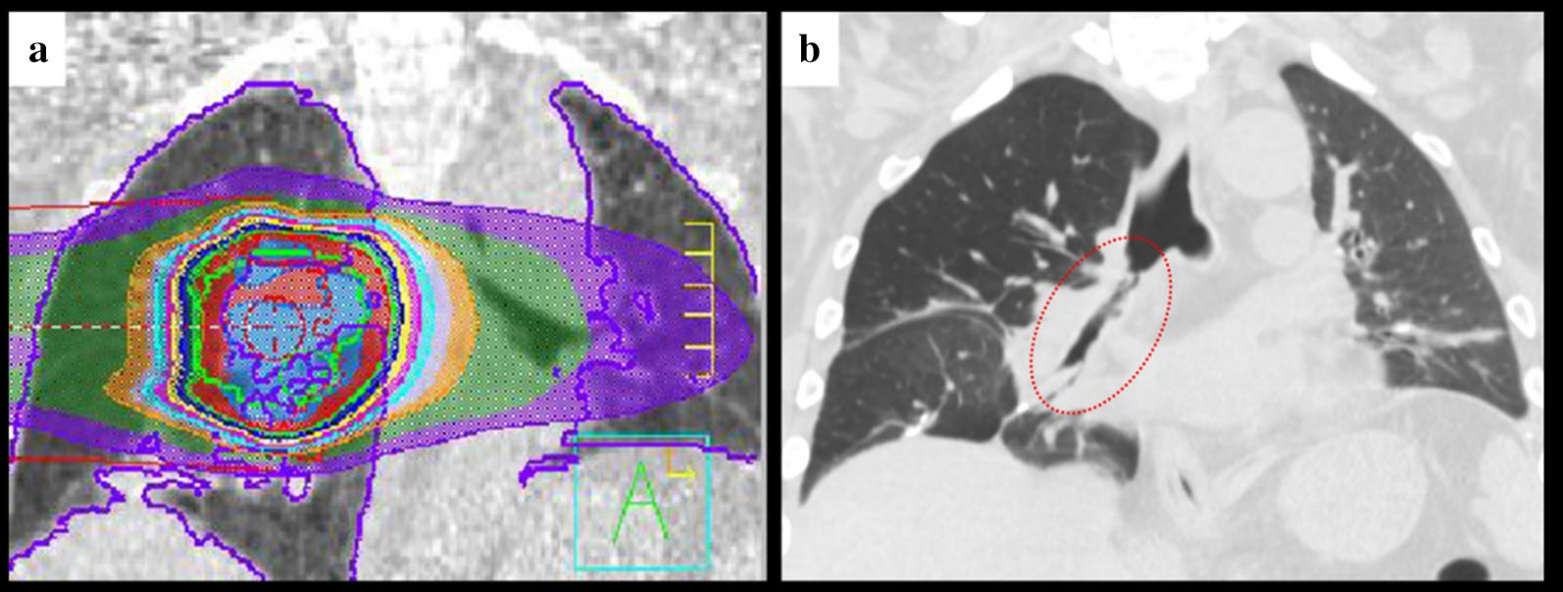
Coronal images of computed tomography before and after radiotherapy. **a** Radiation range shown with a coronal plane. **b** A coronal view showing the wide range of bronchial stenosis, indicating right bronchial wall thickening and narrowing of the bronchial lumen (red interrupted line)

**Fig. 3:**
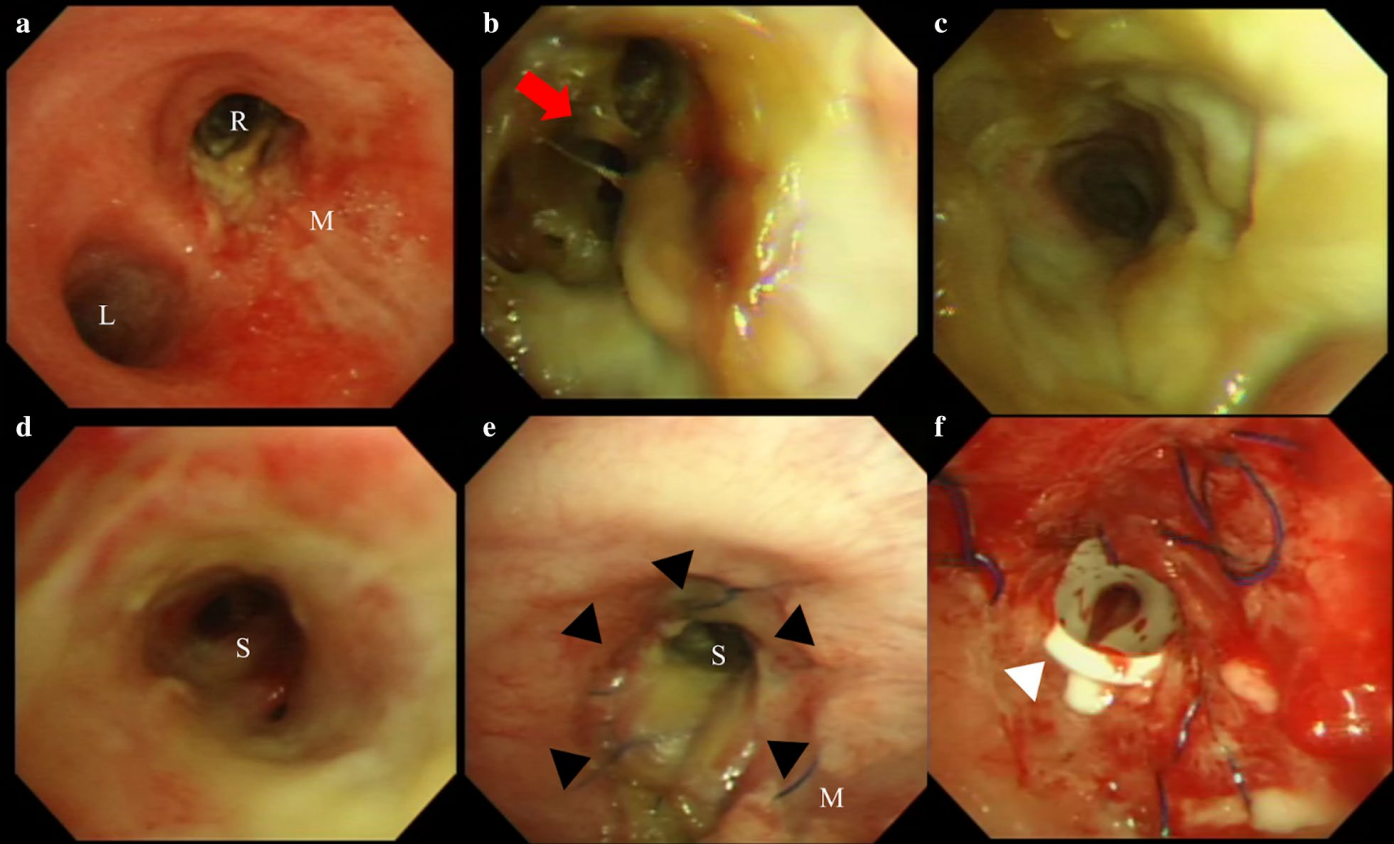
**a–d** Preoperative bronchoscopy after debridement and **e**, **f** postoperative bronchoscopy **a** The trachea, carina, and right (R) and left (L) main bronchus. The tracheal mucosa (M) is inflamed but maintained. **b** The right main bronchus and the secondary carina (red arrow). The bronchial wall is severely necrotic. **c** The bronchus intermedius. The bronchial wall appears necrotic. **d** The lumen of the basal segmental bronchus (S). The mucosa is inflamed but not necrotic. **e** The anastomosis of the bronchus immediately after the operation (black arrowhead). **f** A silicone stent was placed into the stenosis site (white arrowhead)

A 46-year-old man with a smoking history of 15 pack-years presented with cough and dyspnea. He had been treated for a malignant mediastinal germ cell tumor, the original part of which had been surgically resected with part of the left lung and the left phrenic nerve 10 years earlier, resulting in paralysis of the left diaphragm. He had also undergone treatment for bilateral iliac metastasis with chemotherapy and heavy ion radiotherapy 5 years before and metastasis to the right lung with surgical wedge resection 6 years before. Nine months before the outpatient visit, the patient had undergone stereotactic body-radiation therapy with 56 Gy of radiation in seven fractions to treat another right lung metastasis measuring 3.5 cm in diameter and located close to the right hilum (Fig. 1a, b). On presentation, computed tomography revealed right bronchial stenosis over the range of previous radiotherapy (Figs. 1b, c; 2a, b). Bronchoscopy showed broad necrosis of the right bronchus, ranging from the right main bronchus to the orifices of the bronchi of the middle lobe and the superior segment of the lower lobe (Fig. 3a–d). Pulmonary function was low with 2.6 L of vital capacity and 1.7 L of forced expiratory volume in one second. He was repeatedly treated with bronchial debridement of the necrotic tissue, culture of which revealed infection with *Aspergillus fumigatus* and other various bacteria. Bronchoscopic biopsies were performed several times, showing no malignancy but only fungal hyphae. Despite intensive antibiotic therapy with voriconazole and repeated debridement, the necrosis was progressive. Neither balloon dilatation nor stenting seemed to be an option because of the fragility of the bronchial wall and serious concern regarding formation of a bronchopulmonary arterial fistula. The patient eventually developed sepsis and renal failure, which necessitated urgent surgical removal of the necrotic bronchus. However, he was unable to endure right pneumonectomy because of his low respiratory function caused by left phrenic nerve paralysis. Preoperative pulmonary perfusion scintigraphy using technetium-99m-labeled macroaggregated albumin showed moderate-to-severe decrease of blood flow into right upper lobe, and flow volume into right lung was 47%. We therefore performed right lung autotransplantation with preservation of the right basal segment, the orifice of which appeared to still be viable (Fig. 3d).

**Fig. 4:**
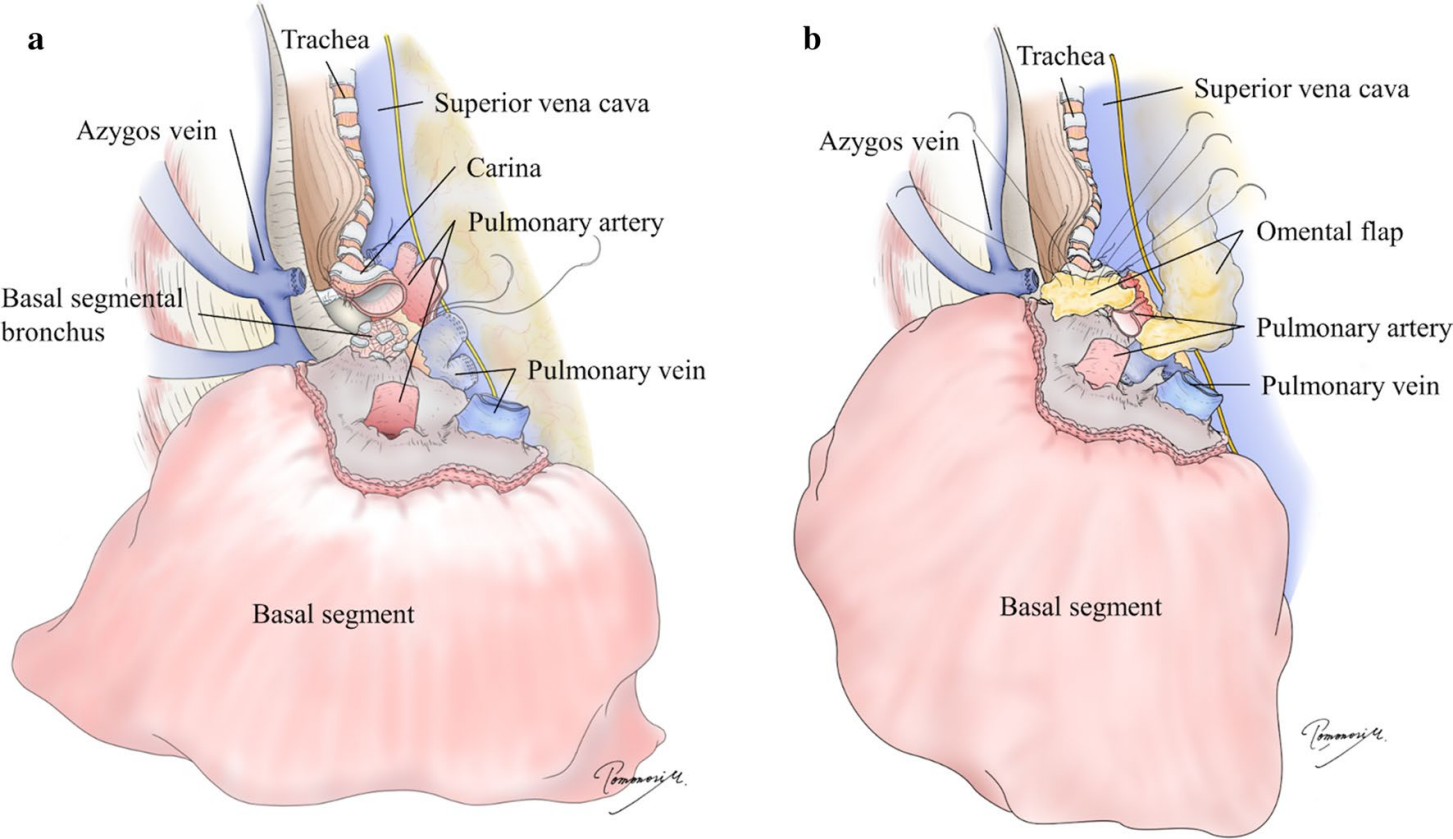
Surgical illustration showing autotransplantation of the basal segment. **a** Anastomoses of the basal segmental bronchus to the carina, the basal pulmonary artery to the right main pulmonary artery, and the basal pulmonary vein to the superior pulmonary vein. **b** Wrapping of the bronchial anastomosis with the omental flap

During the surgery, we first prepared a pedicled omental flap through an upper median laparotomy in the supine position. We continued the surgery in the left decubitus position, and right lateral thoracotomy was performed through the fourth intercostal space. Right pneumonectomy was performed with staplers placed on the right main pulmonary artery and right pulmonary vein, while the right main bronchus was trimmed to the carina because of the broad bronchial necrosis. On the back table, we removed the upper and middle lobes and the superior segment of the lower lobe, then flushed the remaining basal segment anterogradely and retrogradely using extracellular-type trehalose-containing Kyoto (ET-Kyoto) solution in the same manner as in our living-related lung transplantation protocol. Next, we implanted the basal segment by direct anastomosis of the bronchus to the carina, followed by end-to-end anastomoses of the pulmonary arteries and anastomosis of the basal pulmonary vein to the superior pulmonary vein (Fig. 4a). The arterial anastomosis was technically challenging because of the distance and extensive dissection of the main pulmonary artery centrally behind the superior vena cava was necessary, while the venous anastomosis was easy after dissecting the pericardium around left atrium in the same manner as regular lung transplantation. We also wrapped the bronchial anastomosis with an omental flap to facilitate bronchial healing and lower the risk of a bronchopulmonary arterial fistula (Fig. 4b). The operation took about 18 h due to extensive tight adhesions derived from the previous treatment, refractory bleeding from adhered regions and anastomosis of pulmonary artery, and repetitive bilateral lungs ventilation. The back table surgery took about 2 h and graft ischemic time was around 5 h. After operation, the bronchial mucosa of the transplanted basal segment appeared pale distal to the bronchial anastomosis (Fig. 3e).

On postoperative day 9, the patient developed left-sided pneumonia and required temporal support with extracorporeal membrane oxygenation for 2 weeks. One month later, minor bronchial dehiscence at the bronchial anastomosis was observed and was successfully treated with antibiotics and nutritional support. Three months after the operation, the right bronchial anastomosis showed stenosis due to overgrowth of granulation tissue, which was treated with repeated bronchoscopic dilatation and eventual placement of a silicone stent (Fig. 3f). The patient was discharged home 5 months after the operation in a stable condition. At discharge, his pulmonary function was even lower with 1.26 L of vital capacity and 0.88 L of forced expiratory volume in 1 s although he was free of supplementary oxygen.

Three months after discharge, however, the patient developed massive hemoptysis. He was resuscitated on venoarterial extracorporeal membrane oxygenation, and hemostasis was obtained. Because he had fully recovered from the condition, we started discussing further surgical intervention to segregate the bronchial anastomosis and pulmonary artery. Regrettably, however, he soon developed a second massive hemoptysis episode and died.

## Conclusions

Bronchial necrosis is a late complication ranging from months to years after radiation therapy. The mechanism of this condition is considered radiation vasculopathy, resulting in insufficient blood supply to the adjacent airway [1]. This complication is often followed by bacterial or fungal infection due to lack of normal secretion drainage, which enhances the pathophysiological complexity [1]. Therapeutic options for bronchial necrosis associated with radiotherapy include balloon dilatation with or without stent placement and surgical resection with anastomosis based on the location, range, and severity of the necrosis [2]. The optimal treatment has not yet been established, but surgical procedures are absolutely recommended in the presence of pneumomediastinum [1]. The patient in the present case had no signs of pneumomediastinum, but surgical resection was unavoidable because of the severe and extensive necrosis, resulting in sepsis, renal failure, and a high risk of a bronchopulmonary arterial fistula. In addition, reconstruction and preservation of the lung parenchyma were indispensable with limited pulmonary function due to contralateral diaphragm paralysis. Therefore, lung autotransplantation seemed to be the most suitable treatment option.

Lung autotransplantation is an advanced surgical technique that maximizes the patient’s lung function at the time of pulmonary resection [5–7]. Some reports have described adaptation of lung autotransplantation to hilar lung tumors [8–11]; to our knowledge, however, no reports have described the application of lung autotransplantation to bronchial necrosis. The major challenge in applying lung autotransplantation for bronchial necrosis is the possible occurrence of complications related to bronchial anastomosis, the risk of which would generally be much higher than with autotransplantation for hilar lung tumors. Preoperative detailed bronchoscopic evaluation to select the most effective sites to suture the bronchus is a key strategy to reduce the risk of anastomotic complications. Exclusion of recurrent malignancy and control of infection as much as possible are also indispensable. In the present case, the patient developed anastomotic dehiscence followed by stenosis, which had indeed been thought to be inevitable at the time of the initial surgery. The basal segmental bronchus appeared to be inflamed but viable before anastomosis; however, probably in combination with bronchial ischemia due to debranching of bronchial artery, bronchial necrosis was progressive, resulting in late formation of stenotic granulation tissue. Indeed, this anticipated complication was successfully managed with preemptive placement of an omental flap, postoperative medical management, and eventual placement of a silicone stent. Despite the aggressive proactive management, the patient eventually died of bronchopulmonary arterial fistula formation, which is feared as a fatal complication after bronchoplasty. The mechanism by which the fistula developed at such a chronic phase remains unclear. Multiple factors could have been involved in the process, including atrophy of omental flap, chronic infection (especially with *Aspergillus*), chronic ischemic change, extension of radiation-induced necrosis, and a mechanical effect by the silicone stent. This extremely challenging case illustrates the possibility of treating radiation-induced central airway necrosis using an advanced surgical technique such as lung autotransplantation while also showing the risk associated with this challenging surgery. To summarize our preparations for postoperative bronchial complications prior to surgery, bronchoscopic evaluation and careful surgical planning including intraoperative omental flap were considered indispensable. For later bronchial complications, necessity to place a silicon stent at the anastomosis was also anticipated. After the episode of hemoptysis, options including placement of a covered stent in the pulmonary artery and re-operation to isolate bronchus and pulmonary artery were discussed, although a stent with an appropriate size was not found in the limited time and the patient did not agree to additional surgery.

In conclusion, we experienced an extremely challenging case of bronchial necrosis after radiotherapy treated with lung autotransplantation. Prior preparations for postoperative bronchial complications seemed to be the key strategy for this procedure.

## Data Availability

The data used in this report are available from the corresponding author on request.

## References

[1] Aerni MR, Parambil JG, Allen MS, Utz JP (2006). Nontraumatic disruption of the fibrocartilaginous trachea: causes and clinical outcomes. Chest.

[2] Alraiyes AH, Alraies MC, Abbas A (2013). Radiation-associated airway necrosis. Ochsner J.

[3] Corradetti MN, Haas AR, Rengan R (2012). Central-airway necrosis after stereotactic body-radiation therapy. N Engl J Med.

[4] Kim IA, Koh HK, Kim SJ, Yoo KH, Lee KY, Kim HJ (2017). Malignant tracheal necrosis and fistula formation following palliative chemoradiotherapy: a case report. J Thorac Dis.

[5] Chen F, Takahagi A, Sakamoto K, Date H (2015). Lung autotransplantation technique for postpneumonectomy-like syndrome. J Thorac Cardiovasc Surg.

[6] Ding JY (2015). Autotransplantation: a novel solution for postpneumonectomy-like syndrome. J Thorac Cardiovasc Surg.

[7] Emmanouilides C, Tryfon S, Baka S, Titopoulos H, Dager A, Filippou D (2015). Operation for preservation of lung parenchyma in central lung cancer–in vivo and ex situ reimplantation techniques. Anticancer Res.

[8] Oto T, Kiura K, Toyooka S, Miyoshi S (2012). Basal segmental auto-transplantation after pneumonectomy for advanced central lung cancer. Eur J Cardiothorac Surg.

[9] Mao W, Xia W, Chen J, Zheng M (2013). Successful lung autotransplantation for central non-small-cell lung cancer: report of a case. Surg Today.

[10] Watanabe Y, Sato M, Nakamura Y, Hoshikawa Y, Harada A, Nagata T (2015). Right lower lobe autotransplantation for locally advanced central lung cancer. Ann Thorac Surg.

[11] Yamashita T, Hamaji M, Nakanobo R, Aoyama A, Chen-Yoshikawa TF, Sonobe M (2019). Ex vivo sleeve lobectomy and autotransplantation after chemoradiation. Ann Thorac Surg.

